# Training machine learning models with synthetic data improves the prediction of ventricular origin in outflow tract ventricular arrhythmias

**DOI:** 10.3389/fphys.2022.909372

**Published:** 2022-08-12

**Authors:** Ruben Doste, Miguel Lozano, Guillermo Jimenez-Perez, Lluis Mont, Antonio Berruezo, Diego Penela, Oscar Camara, Rafael Sebastian

**Affiliations:** ^1^ Department of Computer Science, University of Oxford, Oxford, United Kingdom; ^2^ Computational Multiscale Simulation Lab (CoMMLab), Department of Computer Science, Universitat de Valencia, Valencia, Spain; ^3^ Physense, BCN Medtech, Department of Information and Communication Technologies, Universitat Pompeu Fabra, Barcelona, Spain; ^4^ Arrhythmia Section, Cardiology Department, Cardiovascular Clinical Institute, Hospital Clínic, Universitat de Barcelona - IDIBAPS, Barcelona, Spain; ^5^ Cardiology Department, Heart Institute, Teknon Medical Center, Barcelona, Spain

**Keywords:** machine learning, electrophysiological simulations, outflow tract ventricular arrhythmias, synthetic databases, virtual population, digital twin

## Abstract

In order to determine the site of origin (SOO) in outflow tract ventricular arrhythmias (OTVAs) before an ablation procedure, several algorithms based on manual identification of electrocardiogram (ECG) features, have been developed. However, the reported accuracy decreases when tested with different datasets. Machine learning algorithms can automatize the process and improve generalization, but their performance is hampered by the lack of large enough OTVA databases. We propose the use of detailed electrophysiological simulations of OTVAs to train a machine learning classification model to predict the ventricular origin of the SOO of ectopic beats. We generated a synthetic database of 12-lead ECGs (2,496 signals) by running multiple simulations from the most typical OTVA SOO in 16 patient-specific geometries. Two types of input data were considered in the classification, raw and feature ECG signals. From the simulated raw 12-lead ECG, we analyzed the contribution of each lead in the predictions, keeping the best ones for the training process. For feature-based analysis, we used entropy-based methods to rank the obtained features. A cross-validation process was included to evaluate the machine learning model. Following, two clinical OTVA databases from different hospitals, including ECGs from 365 patients, were used as test-sets to assess the generalization of the proposed approach. The results show that V2 was the best lead for classification. Prediction of the SOO in OTVA, using both raw signals or features for classification, presented high accuracy values (>0.96). Generalization of the network trained on simulated data was good for both patient datasets (accuracy of 0.86 and 0.84, respectively) and presented better values than using exclusively real ECGs for classification (accuracy of 0.84 and 0.76 for each dataset). The use of simulated ECG data for training machine learning-based classification algorithms is critical to obtain good SOO predictions in OTVA compared to real data alone. The fast implementation and generalization of the proposed methodology may contribute towards its application to a clinical routine.

## 1 Introduction

In structurally healthy hearts, ventricular tachycardia (VT) occurs primarily as a consequence of abnormal ectopic foci in the ventricles, overtaking sino-atrial activation and leading to premature ventricular complexes. The most common type of idiopathic ventricular arrhythmias originates from the outflow tract, and shows a high incidence in young population (Sirichand et al., 2017). For this group of patients, a catheter ablation of the tissue that triggers the ectopic focus is indicated, which shows low procedural complications and a high success rate. However, it is key to previously determine the site of origin (SOO) of the outflow tract ventricular arrhythmia (OTVA) to plan the intervention and the catheter approach. In particular, the differentiation between left and right ventricular (LV and RV, respectively) origin is crucial for the electrophysiologist, being the involved risk and time greatly different.

It is common to obtain recordings of the focal VT in the form of an electrocardiogram (ECG) prior to a radiofrequency ablation (RFA) procedure, which contain important information related to the OTVA and its origin. It is known that the majority of OTVAs originates from the RVOT (70–80%) (Srivathsan et al., 2005). Clinicians have developed several algorithms based on manual feature detection from ECGs (Lerman, 2015) to help determine the SOO. For a review on the classical ECG signatures proposed to determine the SOO of OTVAs, see Anderson et al. (2019). One of the main drawbacks of traditional ECG features is that they are complex to implement in daily clinical practice due to the large number of specific rules that have to be checked, which entail detailed measurements and visual comparison between precordial transitions, signal notches, and other features. In addition, they are usually based on observations on small cohorts of patients from a single-center study. In consequence, the whole process is too dependent on the clinician’s experience. We recently showed that patient-specific simulations can reproduce the ECG signatures of OTVA, being able to predict the SOO in a small cohort of patients from a single center (Doste et al., 2020). However, processing patient data and performing patient-specific simulations requires very specific expertise and it is very time consuming, limiting its implementation in clinical routine.

We propose the application of machine learning (ML) techniques on ECGs from OTVA patients to guide their treatment. The use of ML and deep learning (DL) algorithms to learn from ECG data and provide predictions is becoming very popular in the medical field (Attia et al., 2021; Nagarajan et al., 2021). One particularly successful application is the use of ML for ECG analysis of cardiac arrhythmias, as recently reviewed in Minchole et al. (2019). For instance, ML was applied to classify different types of ventricular arrhythmias by a combination of support vector machine (SVM), with the help of grid search, and waveform morphological analysis (Li et al., 2022). ML has also being used to predict the LVOT versus RVOT SOO of VT in a clinical database of 420 patients with a high accuracy (Zheng et al., 2021), and to localize premature ventricular complexes from ECG using simulated databases (Yang et al., 2017; Alawad and Wang, 2019). Beyond patient stratification, DL has also been used for risk prediction of drug-induced arrhythmias and diagnosis of long QT syndrome (Prifti et al., 2021), or for finding an optimal lead subset of the 12-lead ECG to eliminate the redundancy, improving the generalizability of DL-based models (Lai et al., 2021).

ML and DL techniques rely on the quality of training datasets, which should represent the target population and be balanced. In the particular case of OTVAs, there are different locations for the SOOs (transmurally distributed in several anatomical regions of the LV and RV), and other co-variables such as the ventricular anatomy, its orientation, or the presence of pathological tissue (scar) that affect the ECG morphology. Since there are not large public labelled databases of OTVA patients available (largest in the order of 350 cases), the solution could be the use of computational models, e.g., digital twins (Corral-Acero et al., 2020) to build large virtual datasets where all the variables are under control. These virtual hearts are electrophysiological twins to the patient’s heart on which various stimulation protocols can be applied to, for instance, in our case induce OTVAs from different SOO. Such an approach has been successfully applied to several medical applications, such as drug screening (Costabal et al., 2019), anatomical modelling of pathological populations (Romero et al., 2021; O’Hara et al., 2022), therapy planning of catheter ablation (Ferrer et al., 2015; Ferrer-Albero et al., 2017; Prakosa et al., 2018; Lopez-Perez et al., 2019), or ECG simulation (Cardone-Noott et al., 2016).

In this paper, we propose the use of ML models trained with large synthetic datasets of simulated ECG data obtained from biophysical electrophysiology simulations of OTVAs on digital twins. We present results on the prediction of SOO using different approaches in which we train ML models with a virtual population of synthetic simulated data, validating them with real clinical datasets from different centers, also evaluating combinations of synthetic and real data for training and validation. We also evaluated the best precordial leads and the signal features that produced a better LVOT/RVOT classification. This approach in which all the simulations have been performed beforehand and only ML models are used to predict the SOO of new patients permits its translation to daily clinical routine.

## 2 Materials and methods

**FIGURE 1:**
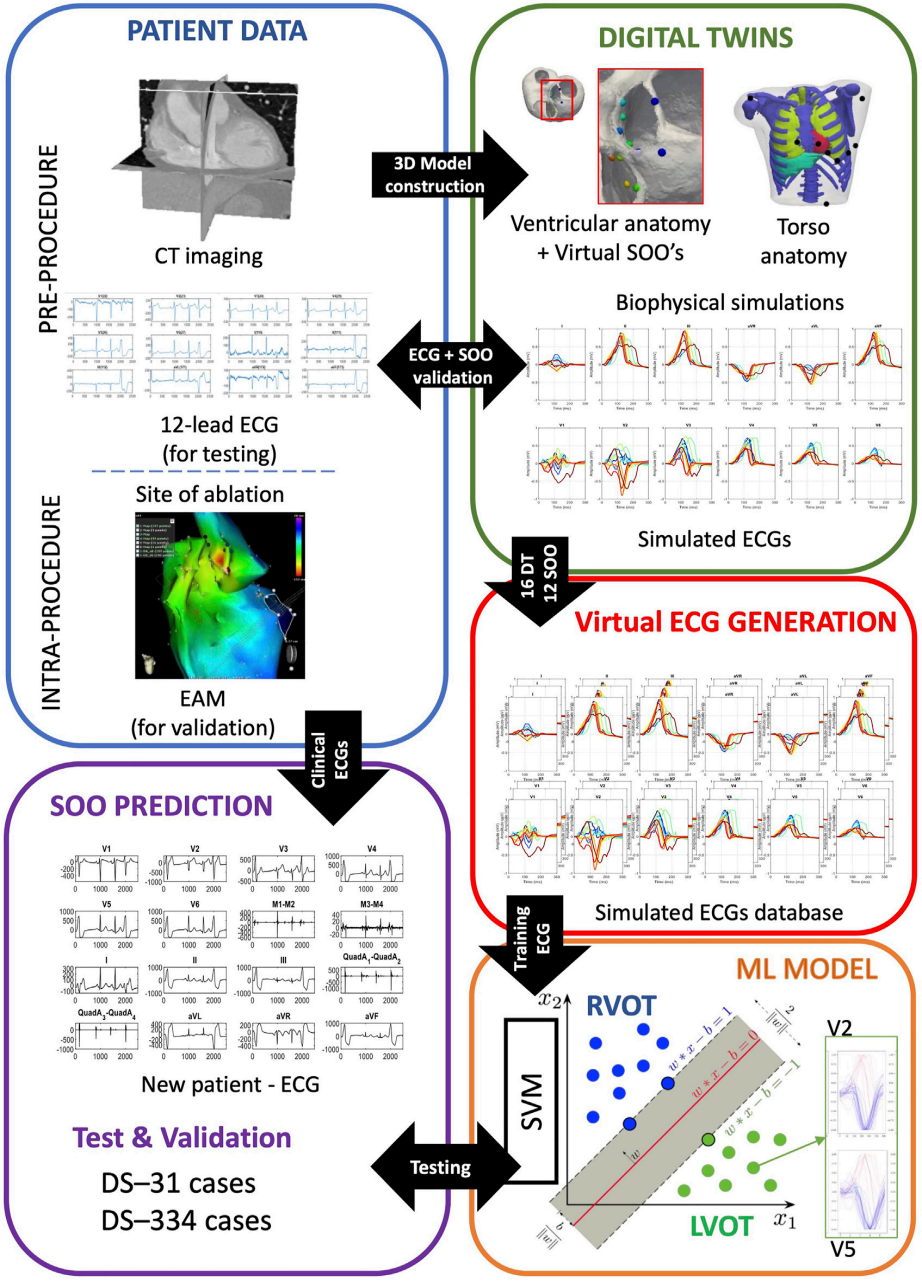
Scheme of the proposed methodology. Patient data was used to build ventricular digital twins for the 16 patients. Biophysical simulations were run in the different anatomies, using 12 different sites of origin (SOO), to generate a database of 12-lead electrocardiograms (ECGs). The database of simulated ECGs was finally used to train a ML algorithm to predict the SOO (Right ventricle outflow tract (RVOT) vs. Left ventricle outflow tract (LVOT)) of real patients ECGs from two different clinical datasets (DS-334 and DS-31). Abbreviations used in the image: CT: computed tomography; EAM: electroanatomical mapping; ML: machine learning; SVM: support vector machine.

We have developed a computational pipeline to build and validate our approach, from clinical data to final ML-based predictions, summarized in Figure 1. It starts with the generation of ventricular digital twins, built with patient-specific heart meshes together with their cellular and tissue electrical properties. All the digital twins include outflow tracts from both ventricles up to the valve planes, with the estimated myocardial fiber orientation as in Doste et al. (2019), and no structural disease. Biophysical simulations of ventricular cardiac electrophysiology were run from different SOO to compute the ECGs. Subsequently, simulated ECGs were compared and validated against the available patient data and used to generate a database of synthetic ECGs. This database was later used to perform a supervised training of a ML model to predict the SOO of an ectopic beat, and was tested against two clinical OTVA ECG databases from different hospitals. In the following subsections we describe the different clinical datasets and the methodology used for simulating the ECGs. The next subsection is then focused on the data pre-processing and homogenization for both clinical and simulated signals. Final subsection contains all the information about the support vector machine used for classification between RV and LV SOO. Two different strategies for reducing the dimensionality of the data (downsampling the raw ECG signal and extracting ECG-based features) are introduced. We also applied a data augmentation algorithm to improve the performance of the classification. Finally, the different scenarios used for the signal classification are described.

### 2.1 Clinical datasets for testing

We included two different clinical ECG datasets from OTVA patients for the validation of the proposed approach. The first dataset consisted of 31 ECGs (DS-31) that were prescribed for catheter ablation procedure at the Hospital Clinic, Barcelona. All patients underwent a electro-anatomical mapping study by CARTO three navigation system (Biosense Webster, Diamond Bar, CA, United States ) with a 3.5 mm irrigated tip catheter (NaviStar, Biosense Webster). During the procedure, 12-lead surface ECG and intracardiac recordings were obtained and displayed by an electrophysiology data acquisition system (Bard LabSystem, CR Bard Inc., Lowell, MA, United States ; or EP-Tracer, CardioTek, Maastricht, Netherlands). Ablation was considered successful if the targeted OTVA was eliminated and it was noninducible after isoproterenol infusion. The site where RFA application eliminated the OTVA was considered the SOO and was labelled and saved in the electroanatomical mapping data for validation purposes. The study was approved by the local ethics committee and written informed consent was obtained from all participants. The second dataset was an open-source 12-lead ECG database of 334 OTVA patients (DS-334) published by Zheng et al. (2020). The database was composed of 257 patients that had arrhythmias originated in the RVOT and 77 patients with an LVOT origin, which were treated at the Ningbo First Hospital of Zhejiang University (China). ECG signals were obtained at a sampling rate of 2 kHz. Details about the RFA procedure, ECG acquisition or ethical committee can be found in the original study (Zheng et al., 2020).

### 2.2 Virtual electrocardiogram generation

In this work, we constructed ventricular digital twins from 16 different biventricular geometries built from patient-specific computed tomography (CT) scans. Each model was represented by a volumetric 3D mesh made of hexahedral elements with an average resolution of 400 *μm*. Every element was labeled according to its cellular properties as, endocardial, midmyocardial or epicardial cells. As described in Doste et al. (2020), for each digital twin, we simulated OTVAs from 12 different SOOs (see Figure 1, digital twins, spheres on biventricular geometry) chosen following clinical observations (Anderson et al., 2019), seven from the LVOT and five from the RVOT.To perform the simulations at the organ level, we used the software ELVIRA (Heidenreich et al., 2010), which solves the anisotropic reaction-diffusion equation of the monodomain model for cardiac EP using finite element methods. For the numerical solution of our simulations, we applied the conjugate gradient method with an integration time step of 0.02 ms, using implicit integration for the parabolic partial differential equation of monodomain model and explicit integration with adaptive time stepping for ordinary differential equation of the ionic model (ten Tusscher et al., 2004). Each simulation consisted in a train of four beats with a cycle length of 800 ms followed by an ectopic focus simulated during a time window of 300 ms. Extracellular potentials at the heart were approximated from transmembrane potentials previously computed, and propagated by using the finite element method to solve a Laplace equation over the volume mesh of a generic 3D torso model (Lopez-Perez et al., 2019). Torso anatomy included the lungs, ribs, liver, atria and a cavity where each biventricular model was fitted. To add extra variability on the simulated ECG that can be produced by different lead placement or heart orientation, we shifted precordial leads around the standard position to have 13 different lead configurations. Consequently, we built a database of a total of 2,496 12-lead simulated ECGs (16 patients, with 12 different SOO and with 13 different electrode placements). ECGs were validated against patient data using the 12 lead correlation coefficient and the LV/RV ratio (Doste et al., 2020). This ratio was calculated by dividing the mean of the 12 lead correlation coefficient values of all the SOO simulations with origin in the LVOT by the one corresponding to the SOO simulations originated in the RVOT. A LV/RV ratio larger and smaller than one will indicate a LVOT and RVOT origins, respectively.

### 2.3 Data pre-processing

We performed data homogenization to facilitate the data processing by the ML algorithm and a better comparison of the results. In particular, we classified all the ECGs (real and simulated) according to the SOO provided in the work by Zheng et al. (2020). All the OTVA ECGs were divided in two main groups as a function of the origin: LVOT or RVOT. The SOO were also distributed in different sublocations. LVOT cases were classified into six regions: Left coronary cusp (LCC), right coronary cusp (RCC), LCC-RCC commissure, non-coronary cusp (NCC), aortomitral continuity (AMC) and LV epicardial summit. RVOT cases were divided in anteroseptal RV, posteroseptal RV and right ventricular free wall (RFW). These regions can be visualized in the geometry shown in Figure 4. Only the QRS complex of the ECGs was evaluated. To standardize the input to the ML models, each 12-lead ECG amplitude was max-abs normalized (i.e., normalized in the range [-1, 1]) and the onset and offset of the QRS complexes were obtained using a DL-based ECG delineator (Jimenez-Perez et al., 2021a; Jimenez-Perez et al., 2021b) for posteriorly using them in raw- and feature-based approaches (Section 2.4). In feature-based approaches, the signal was further zero-corrected to remove baseline wander, and transformed using the wavelet transform (Martinez et al., 2004) and Welch’s periodogram (Welch, 1967) for the feature extraction pipeline.

### 2.4 Machine learning model

We chose support vector machines (SVM) to classify patient arrhythmias as a function of the SOO. An SVM is a well-known learning algorithm (Cristianini and Shawe-Taylor, 2000) that has extensively been used in many clinical areas, such as ECG classification (Attia et al., 2021), due to a remarkably robust performance when working with sparse and noisy data. SVMs tries to separate a given labeled training set (LVOT vs. RVOT origin) with a hyper-plane that is maximally distant from them. In our case, we use radial basis function kernels that will produce non-linear decision boundaries. We have applied two strategies for reducing the dimensionality of the data used for model training, since high dimensionality directly affects the classification performance by introducing unwanted noise. These strategies included downsampling the raw ECG signal and using this morphology directly (Section 2.4.1) and extracting ECG-based features (Section 2.4.2). The final number of features and samples in the down-sampled signal was chosen by evaluating the cumulative variance against the number of principal components of the training signals. We also evaluated the information carried in each lead by analyzing the classification performance of using specific lead combinations.

#### 2.4.1 Raw signals

**FIGURE 2:**
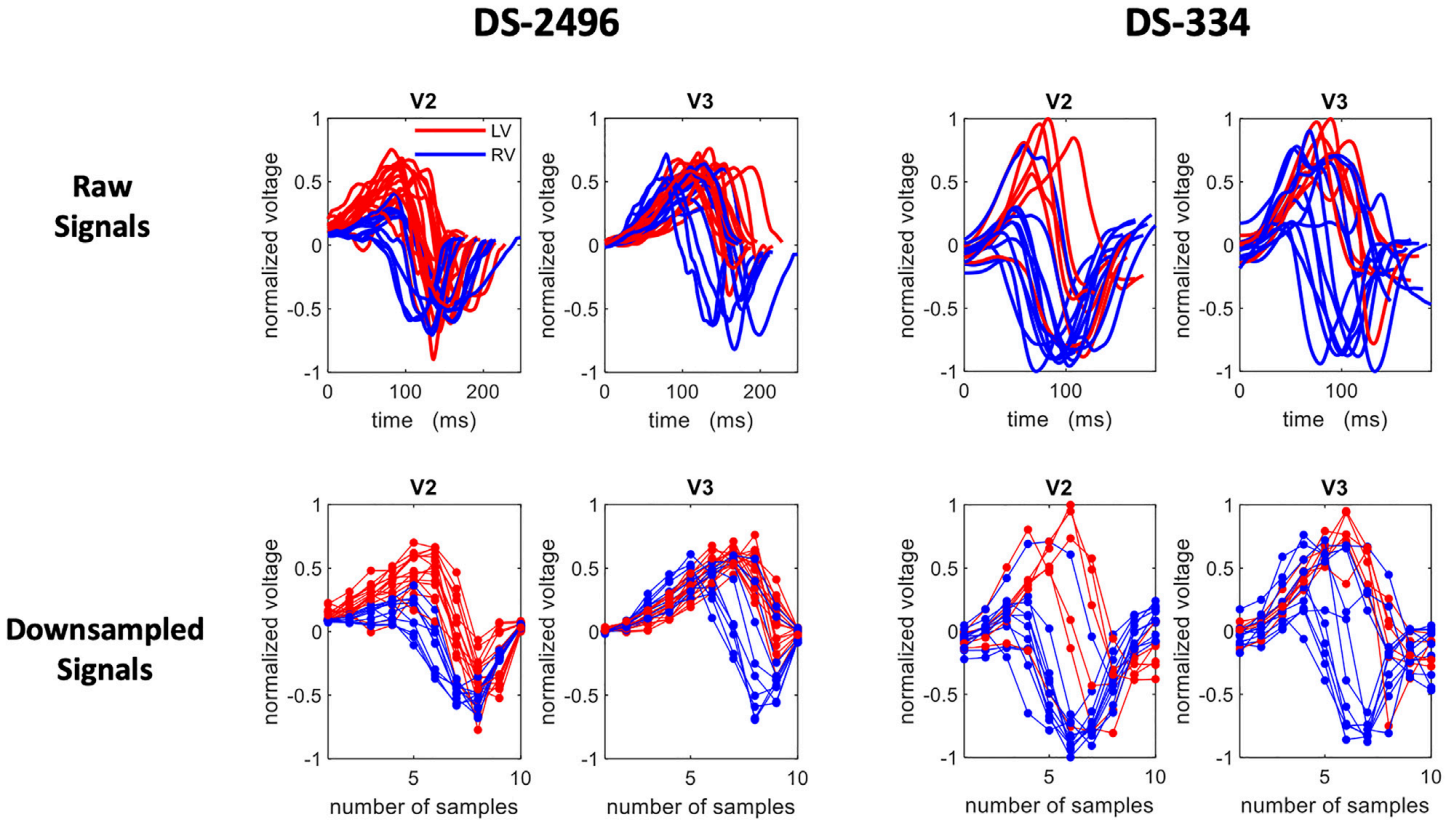
A set of selected ECG traces from the simulated (DS-2496) and the DS-334 datasets. The extracted QRS of the V2 and V3 precordial leads are shown in the top row and their corresponding down-sampled traces (10 samples) in the bottom row. Right Ventricle (RV) signals are plotted in blue, whereas left ventricle (LV) signals are in red.

Since all ECG signals were conveniently normalized and aligned, segmented QRS complexes could be directly treated as patterns and also as feature vectors, where the pseudo-features correspond to the ECG amplitude at each time point. Given that the changes in the voltage convey the most important information of the ECG, we simply consider that the down-sampled raw signal (dimensionality reduction) is a set of features that represent the data at specific time points around the R-peak. Therefore, after studying the cumulative variance of the principal components, the signals were down-sampled to 10 samples (see Figure 2).

We used this down-sampled raw signal representation to determine the best lead combinations as well as the most important lead in terms of classification. This exploration was carried out exhaustively, that is, for each one of the possible lead combinations (4,095). Therefore, a SVM classification model was trained with the corresponding lead combinations and then evaluated with the two test datasets. To obtain the feature vector of a lead combination we simply concatenate the corresponding signal leads (increasing the number of dimensions associated to the classification tasks). Finally, we computed the accuracy distribution associated to each lead by considering all the accuracy obtained from any lead combination that contain that particular lead.

The signal low dimension representation can also be used to determine what part of the signal is the most important in terms of classification. To assess this issue, we calculate a feature importance ranking based on *extra* − *trees* classifier models. In this kind of forests, the importance of the features are computed as the mean and standard deviation of accumulation of the impurity decrease within each tree (entropy based) and it is provided by the fitted model. The feature importance is specially interesting for raw signals as each feature covers a short time interval of the beat, so that the most important features correspond with the time intervals used by the classifier that better explain its predictions.

#### 2.4.2 Feature-based signals

##### 2.4.2.1 Feature extraction

**FIGURE 3:**
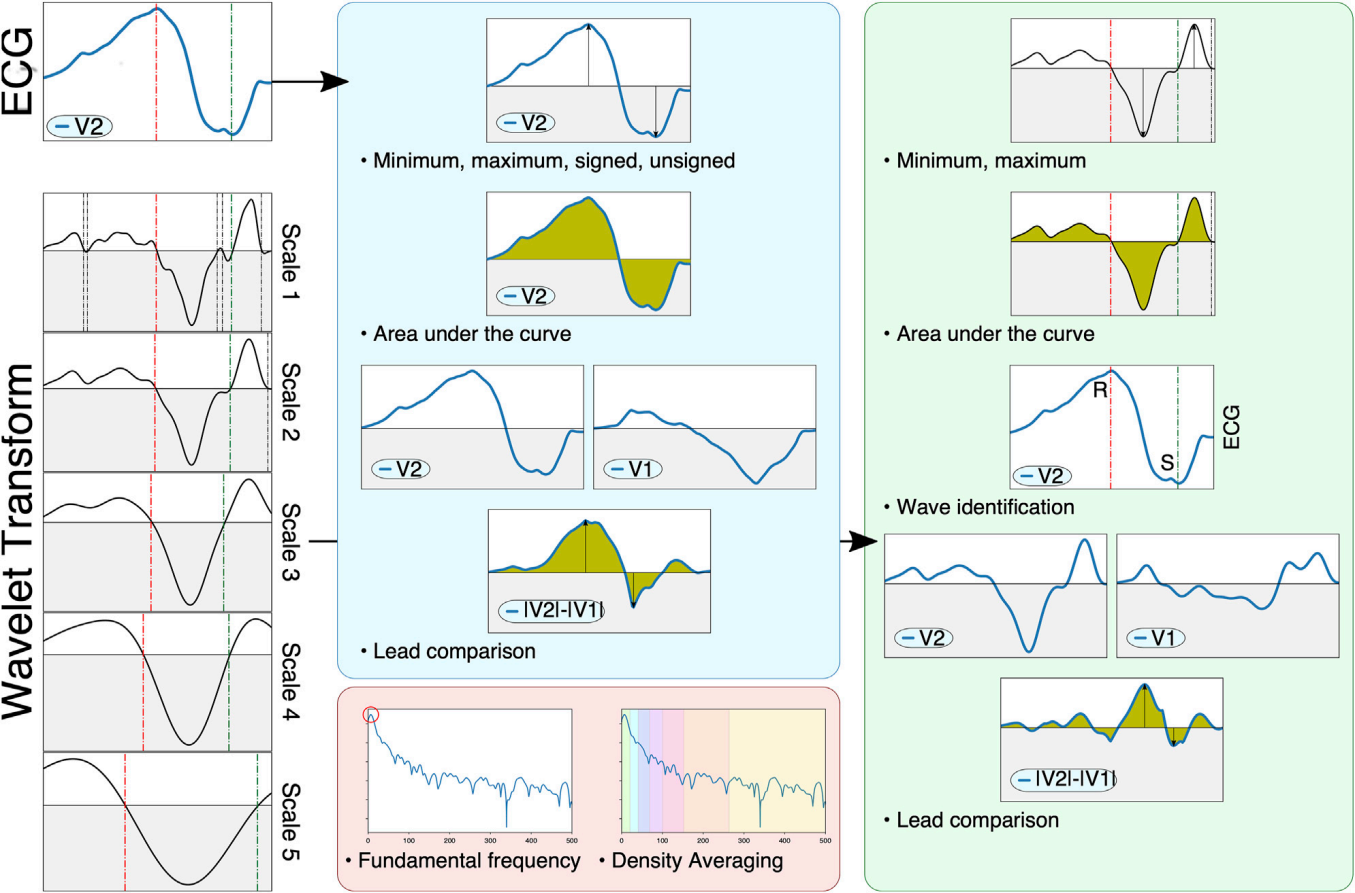
Schematic representation of the feature extraction pipeline. The ECG (left, top) and its wavelet transform (left, scales one through 5) are used to compute signal-based (blue), wavelet-based (green) and spectral-based (red) features. Signal-based features measure the raw ECG, computing extrema, areas or characteristics computed through pairwise lead comparison. Wavelet-based features also compute said markers, but also identify important fiducials through zero-crossings (e.g. different QRS waves such as the R and S waves, shown on the left as green and red dotted lines). Finally, spectral information employs Welch’s periodogram to obtain features based on the signal’s power spectral density.

Previous studies have reported a decrease in generality when using the raw ECG trace as opposed to ECG-derived features (Minchole et al., 2019). To address this, feature extraction was performed on the QRS complex as an alternative representation of its morphology. A total of 356 features were extracted, based on measurements on the raw ECG, its wavelet transform and its power spectral density. Some features were computed using single leads (e.g. maximum of lead V3), whereas other features performed pairwise comparisons of leads (e.g. area under the curve of lead II with respect to lead III). To avoid too high dimensionality, comparative features were only computed within three subsets of leads: limb leads (I, II and III), augmented leads (aVR, aVF and aVL) and precordial leads (V1 to V6), with a total of 21 comparisons per extracted feature. Finally, some features explored the effect of many leads (e.g. precordial transition explores the lead where the polarity changes, taking all precordial leads). Some of the extracted features were inspired in the methodology presented by Maršánová et al. (2017), which provided high accuracy when classifying heartbeat types. A schematic representation of the extracted features is depicted in Figure 3.

The signal-based features consisted in the computation of several markers from the raw ECG. Firstly, two all-lead features were considered: the QRS′ total activation time (QRS_*end*_ − QRS_*start*_) and the localization of the precordial transition. The precordial transition was computed via retrieving the signed maximum of each precordial lead and selecting the first lead where the QRS complex changed polarity, codified as a decimal point value within zero (V1) and one (V6). Secondly, eight per-lead features are extracted. These comprise the polarity of the lead’s signed maximum ({ − 1, 1}), the lead’s maximum and minimum voltage, its absolute maximum voltage (signed and unsigned), the lead’s amplitude and its area (both raw and taking the absolute value). Finally, three comparative features were computed: the signed maximum voltage of the difference between the leads, the area of the difference between the leads and the cross-correlation between the leads.

Wavelet-based features were computed with the mother wavelet designed by Martinez et al. (2004), which has a frequency response that is optimal for QRS complexes. The wavelet transform is used in this work as a robust surrogate of the original signal’s derivatives, and was employed to locate different fiducials in the signal (Q, R and S wave peaks) through the identification of zero crossings across multiple wavelet scales (Figure 3, bottom-left). This allowed a better characterization of important clinical markers such as the ratio between the R and S waves or the maximum signal velocity. Firstly, a single all-lead feature was extracted, the precordial transition, by estimating the moment where the precordial leads changed polarity. Secondly, six per-lead features were computed, comprising the maximum and minimum amplitude values, the mean amplitude of the wavelet, its area under the curve, the signal fragmentation (estimated as the ratio between the wavelet’s area and its absolute area) and the R/S amplitude ratio. Finally, three comparative features were extracted, consisting in the area under the curve, the maximum difference and the cross-correlation between two leads. Although the identification of important fiducials was performed by propagating the information across different wavelet scales, only the first wavelet scale was employed for the above computations.

Finally, seven per-lead spectral features were extracted. For that purpose, the power spectral density of the QRS complex was computed with the method proposed by Welch (1967). After computing the power spectra (Figure 3, red block), the fundamental frequency was estimated by computing the frequency with the highest power, and six spectral density bands were computed by averaging the signal’s power between (0.3) (3.6) (6.9) (9.12) (12.25) and (25.50) Hz.

##### 2.4.2.2 Feature importance

When working with the 356-featured training set, the selection of the best features for classification has been carried out using *extra* − *trees* classifier models (Geurts et al., 2006). The model consists on a meta estimator that fits a number of unpruned randomized decision trees (*extra* − *trees*) on various sub-samples of the training set. Then, predictions are made by majority voting from the trees. Similar methods like bagging and random forest develop each decision tree from a bootstrap sample of the training set, while the *extra* − *trees* algorithm fits each decision tree on the whole training set. Furthermore, similarly to the random forest method, the *extra* − *trees* model will randomly sample the features at each split point of a decision tree. However, random forest uses a greedy algorithm to select an optimal split point, while the *extra* − *trees* model selects a split point at random. All the features extracted from the simulated dataset (DS-2496) were ranked according with the results of the *extra* − *trees* classifier. A variance analysis was also conducted to evaluate the minimum number of features that optimizes the classification performance.

#### 2.4.3 Data augmentation

In order to improve the performance of the classification task, the simulated dataset was augmented using mixup, as described by Zhang et al. (2018). This technique allowed for smoother decision boundaries when training a classifier on augmented data: augmented samples are generated by randomly selecting two samples (**x**_*i*_ and **x**_*j*_) and performing a linear combination of the two $\left(\hat{x}=\lambda x_{i}+\left(1-\lambda \right)x_{j}\right)$ , given a parameter generated by a beta distribution (λ∼B(α,β),λ∈[0,1]) . In the same signal generation process, the corresponding label (*y*_*i*_ or *y*_*j*_) was fixed through the *λ* parameter: *y*_*i*_ was adopted if *λ* > 0.5, and *y*_*j*_ was selected otherwise.

**FIGURE 4:**
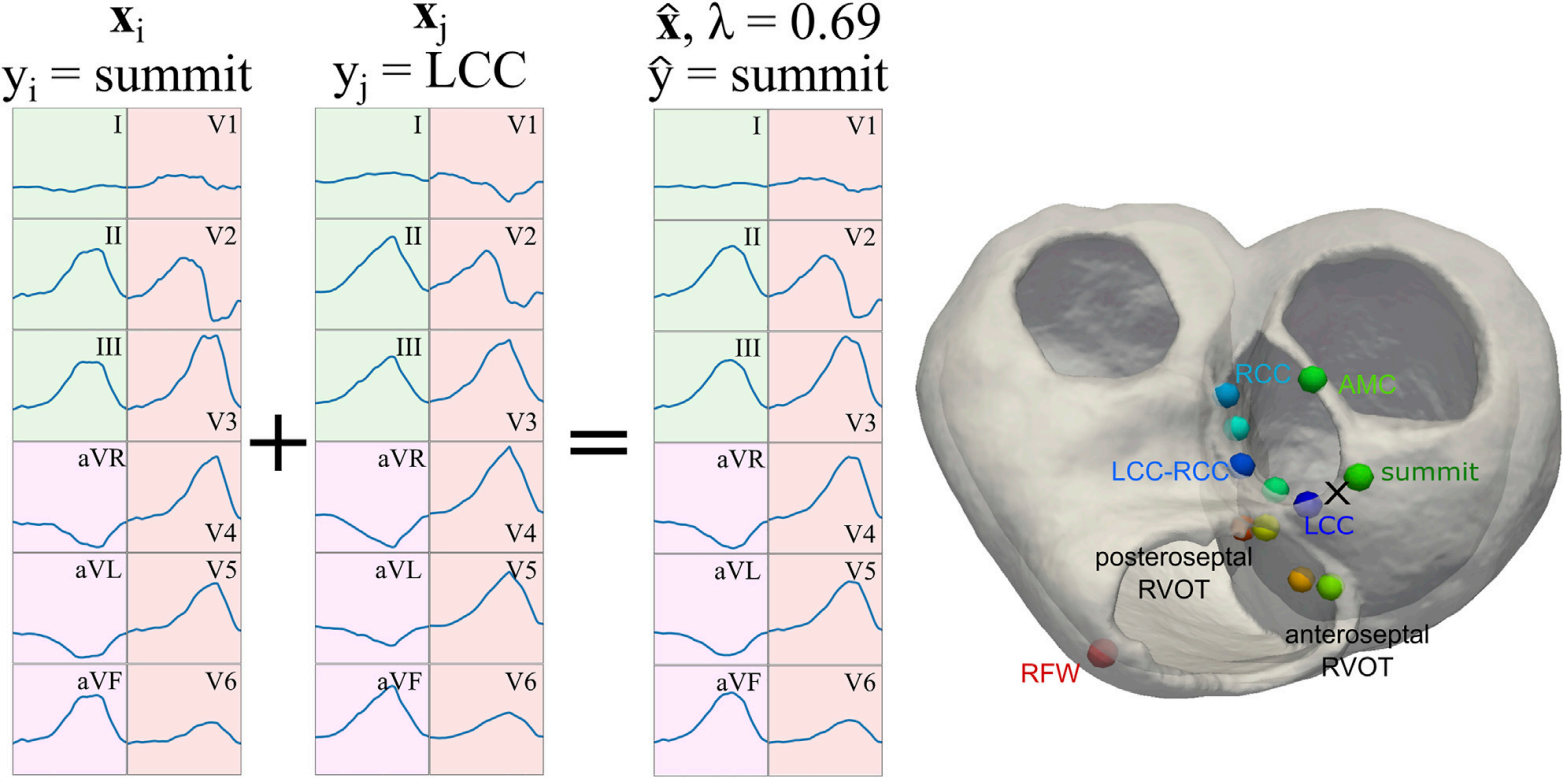
Representation of the signal augmentation process. Two samples **x**_*i*_ and **x**_*j*_ are linearly combined with λ∼B(α,β) . The label of **x**_*i*_ is adopted, given that *λ* >0.5. In the right, marked with a cross, the approximated location of the augmented sample. LCC: left coronary cusp; RCC: right coronary cusp; RFW: right free wall; AMC: aortomitral continuity; RVOT: right outflow tract.

In this work, we employed single 12-lead QRS complexes as samples **x**_*i*_, and the labels *y*_*i*_ were the ground-truth SOO (either LVOT vs. RVOT or the nine finer SOO sublocations). For the purposes of this work, *α* = 5 and *β* = 1.5 were selected as hyperparameters. The *λ* parameter was saved to be used as sample weight in the classification process. In the case of the finer sublocations, and to avoid issues with labels corresponding to distant sublocalizations (e.g. mixing Anteroseptal and AMC SOO samples), mixup was only applied when **x**_*i*_ and **x**_*j*_ were neighboring segments in a spatial sense, as can be seen in Figure 4. Finally, the generated QRS complexes were in turn employed for classification with the raw signal, as described in Section 2.4.1, and with the feature extraction pipeline explained in Section 2.4.2. In total, 7,488 augmented QRS complexes were generated for the virtual ECG population described in Section 2.2. A table with comparison metrics of the different databases, including the augmented database, can be found in the Supplementary Material S1.

#### 2.4.4 Classification and validation

To evaluate the degree of generalization achieved by the SVM models and to exploit the datasets used in this work, we considered the following scenarios (See Table 1):

**TABLE 1:** Description of the different classification scenarios.

Scenario	Training	Classification Strategy
Scenario 1 (Sc1)	SVM model trained with simulated signals (DS-2496) or augmented simulatedsignals (DS-7488)	down-sampled raw signal; feature-based ECG signals; 10 best features
Scenario 2 (Sc2)	SVM model trained with real signals (DS-334; DS-31)	down-sampled raw signal; feature-based ECG signals
Scenario 3 (Sc3)	SVM model trained with a hybrid training set (DS-334) + (DS-2496); (DS-31) +(DS-2496)	down-sampled raw signal; feature-based ECG signals

As we manage four datasets, namely DS-2496 (simulated signals), DS-7488 (simulated augmented signals), DS-334 (real patient signals), and DS-31 (real patient signals), the test-set(s) used for the assessment of each learning scenario are those not employed in training. Furthermore, cross validation (CV) techniques (folds = 5) in the domain of the training set are also included. Accuracy values obtained for the DS-334 and DS-31 datasets were computed as balanced accuracy. More information about the classification of the main Scenarios (confusion matrix and accuracy per class) is attached in the Supplementary Material S1.

## 3 Results

### 3.1 Variance analysis

**FIGURE 5:**
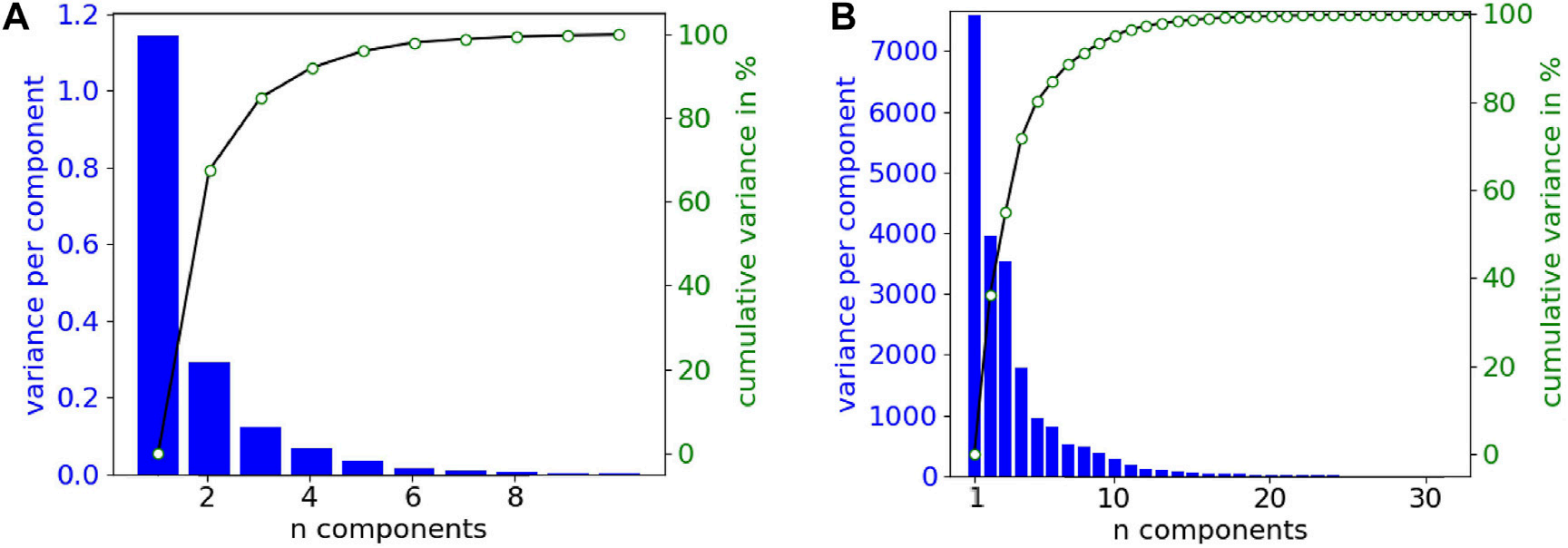
Variance explained by the principal components from the simulated dataset (DS-2496). **(A)** Down-sampled raw signals (10 samples or features). **(B)** Featured signals (356 features).

Figure 5 shows the variance explained by a principal component analysis (PCA) of the simulated dataset (DS-2496) according to the number of components, in the two signal representations handled in this work; raw (a) and featured (b). In both cases, the number of features required to cover almost 100% of the variance is low (10 features). Therefore, the down-sampling of the raw signals allows to easily reduce the number of features of the dataset in similar way to PCA, although in this case the new features are directly related with the electrical potential mean values of time intervals.

### 3.2 Best lead combination for classification with raw data

**FIGURE 6:**
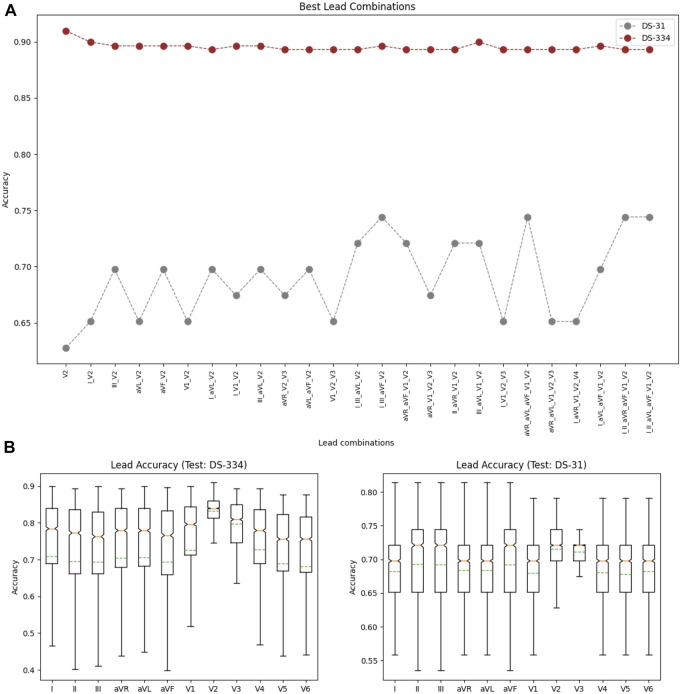
**(A)** Lead combinations that presented the best classification results for both datasets. **(B)** Distribution of the classification accuracies associated to each lead for the DS-334 (left) and DS-31 (right) datasets.

**FIGURE 7:**
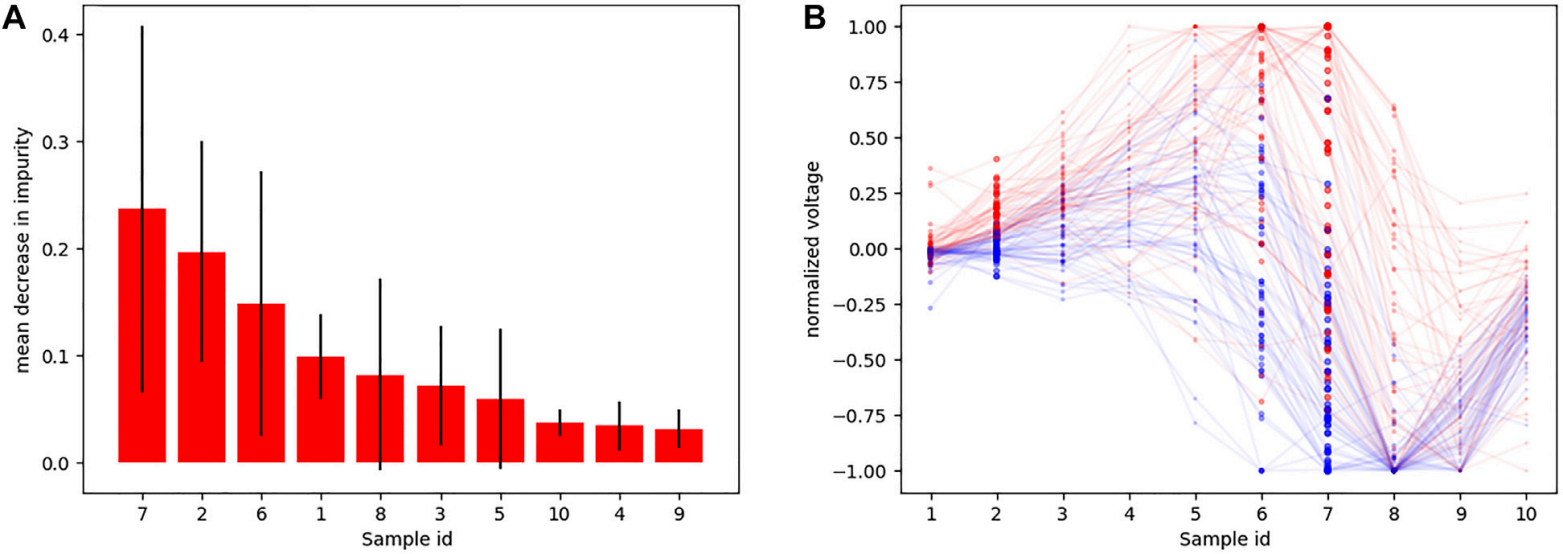
**(A)** Sample importance obtained using raw down-sampled signals; **(B)** Ten samples (red and blue dots) corresponding to signal time intervals obtained from a subset of V2 signals, together with their corresponding traces (transparent) so that they could be easily interpreted. Red traces correspond to LVOT and blue traces to RVOT.

Using the down-sampled simulated raw data (10 equidistant samples from the voltage ECG traces), we explored which were the lead combinations that showed a better performance for ECG classification. A total of 4,095 different models were trained using all the possible lead combinations. Figure 6A shows the best 24 combinations that presented the highest classification results. The accuracy distribution associated to each lead is presented in Figure 6B. Lead V2 is the lead that presents higher accuracy in both testing sets (followed by lead V3), and is also present in all the best lead combinations for DS-334. When comparing the different accuracy obtained with both datasets, DS-31 presents overall lower accuracy values.To uncover the characteristics of V2 that might be responsible for the higher classification accuracy, we evaluated the importance of each samples in the downsampled raw signal. The results are depicted in Figure 7A. The importance of each of the 10 samples is represented by the red bars, being the seventh sample the most important one. Figure 7B depicts a small subset of V2 traces from the LVOT (red) and RVOT (blue) simulations overlaid to the down-sampled signal samples (red and blue dots). Results show that the seventh bin samples, where differences in voltage between LVOT and RVOT simulated traces are clearly seen, are the most important for the classification. The second most important bin is the second, where traces show a positive or negative slope. The adjacent samples to the second and seventh bins continue the order of descending sample relevance.

### 3.3 Feature selection

**FIGURE 8:**
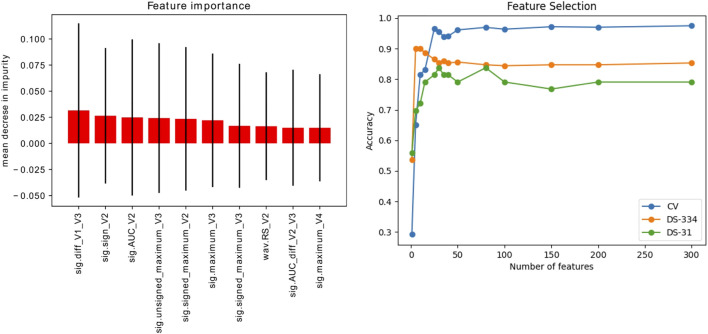
Left: Ranking of the 10 most important signal features (out of 356) for signal classification, extracted using the extra-trees classifier method. Right: Evolution of the accuracy scores vs. the number of features used for training. The accuracy was evaluated in the simulated signals through a cross validation (CV) process and in the clinical datasets (DS-334 and DS-31).

Figure 8A shows the ten most important features obtained using the introduced *extra* − *trees* model. As can be seen, the most important features were signal-based features extracted from the V2 and V3 leads. In addition, the panel on the right shows the accuracy scores obtained by the SVM-classifier by varying the number of features used in the training set. The calculated scores are the accuracy from a cross validation process (folds = 5) and the predictions of the datasets employed for model testing (DS-334, DS-31).

### 3.4 Classification results

Table 2 shows the classification performance of the different scenarios described in the methodology. The cross-validation analysis of the classification with each of the datasets, using five folds, provided high accuracies between 0.82 and 0.96 for the simulated data, and low accuracies for the clinical datasets, that were between 0.60 and 0.92. DS-31 was particularly complex to classify due to its reduced size and high data variability.

**TABLE 2:** SVM accuracy results.

Scenario	Training Set	Accuracy		
		CV (folds = 5)	Test (DS-334)	Test (DS-31)
Sc1	Simulated Raw Signals (DS-2496)	0.96	0.86	0.71
Sc1	Augmented Simulated Raw Signals (DS-7488)	0.98	0.86	0.71
Sc1	Featured Simulated Signals (DS-2496)	0.97	0.85	0.84
Sc1	Featured Augmented Simulated Signals (DS-7488)	0.98	0.86	0.84
Sc1	10 Best Features Simulated Signals (DS-2496)	0.82	0.88	0.77
Sc1	10 Best Features Augmented Simulated Signals (DS-7488)	0.87	0.86	0.77
Sc2	Real Raw Signal (DS-334)	0.88	-	0.57
Sc2	Real Featured Signal (DS-334)	0.92	-	0.76
Sc2	Real Raw Signal (DS-31)	0.62	0.84	-
Sc2	Real Featured Signal (DS-31)	0.60	0.74	-
Sc3	Hybrid: Simulated + Real Raw Signals (DS-334)+(DS-2496)	0.90	-	0.71
Sc3	Hybrid: Simulated + Real Featured Signals (DS-334)+(DS-2496)	0.96	-	0.81
Sc3	Hybrid: Simulated + Real Raw Signals (DS-31)+(DS-2496)	0.95	0.86	-
Sc3	Hybrid: Simulated + Real Featured Signals (DS-31)+(DS-2496)	0.97	0.85	-

Scenario 1, where only simulated signals were considered for training, presents the highest accuracy values in the classification. Raw down-sampled signals were able to classify the clinical signals of the DS-334 with an accuracy of 0.86, whereas the DS-31 presented lower values (0.71). Results did not improve significantly when we made use of augmented simulated data for training (DS-7488). The use of signal features also presented good classification values. Although the accuracy slightly decreased in the DS-334 with respect to the raw data (0.85 vs. 0.86, featured-based vs. raw data), the accuracy for dataset DS-31 was considerably higher (0.84 vs. 0.71, featured-based vs. raw data). As with the case of raw data, augmented feature data barely increased the accuracy values. Furthermore, as shown in the cumulative variance plot, classification with only 10 features was also performed. Results showed that a classification using the best 10 features, determined in Figure 8, provided good accuracy values, although the cross-validation accuracy values were slightly lower than the ones obtained using all the features (356).

Scenario 2, which was calculated using only real data for training, served as a good comparison of the classification performance of the simulated data versus the real data. It can be seen that, when using signals from different clinical datasets for training and classification, the accuracy in the prediction of the SOO decreases significantly, being inferior to the values of Scenario one in all cases. Scenario 3, on its behalf, used a mix of simulated and real data for training. All the accuracies (using raw data and featured data) surpassed the values of the Scenario 2, showing that the use of simulated data in the training can considerably improve the classification results.

## 4 Discussion

In this work, we have presented a methodology to automatically classify ECGs from patients that suffer OTVAs by a ML model purely trained with synthetic data from biophysical simulations carried out on ventricular digital twins. To this end, we trained SVMs classifiers that were able to determine the SOO of the arrhythmia, differentiating between LVOT and RVOT. We have validated the method with two clinical datasets acquired in different clinical centers. In particular, we show that this method can predict the SOO with an accuracy of 0.86 in a clinical database of 334 patients, and 0.84 in a second clinical database of 31 patients, without the need of performing any manual analysis on the ECG signals. This is key, since other algorithms in the literature require an electrophysiologist to extract a considerable number of the features from the 12-lead ECG signals and perform several calculations on them to predict the SOO (Anderson et al., 2019). Further, we have been able to show that a ML model for ECG classification can be trained on virtual ECGs, eliminating the need to collect and curate large clinical databases (Zheng et al., 2020). Another advantage of this simulation strategy is that the signals are noise free, and the location of the SOO is determined without any error in position. Finally, the dataset built to train the ML model can include a balanced number of samples that represent properly all the SOOs, and possible variations of the heart with respect to the torso, such as rotations, which is really complex to achieve with clinical data due to the incidence of the pathology in the population (70% of cases correspond to RVOT SOO) (Srivathsan et al., 2005).

Our analysis on the use of different combinations of signals to train the model and predict the SOO pointed out that V2 was the signal that convey more information followed by V3. This is in agreement with the results already reported in a few clinical studies (Hayashi et al., 2017; Kaypakli et al., 2018). A more exhaustive evaluation of the down-sampled version of the V2 lead, showed the most important signal samples used for the classification by the SVM. In particular, these positions, usually located after the R peak, corresponded with the signal parts that presented higher variability in voltage between RVOT and LVOT (once these signals have been aligned and normalized).

The obtained classification results had a similar level of accuracy than clinical algorithms used in the SOO prediction (Anderson et al., 2019; Mariani et al., 2021). From these results, we have been able to conclude that, although extracting signal features from ECG seems to be the best approach, there is not a large improvement with respect to simply use raw data as features (the potential of the signal at ten equally spaced time points), provided that all signals are aligned. That means that, if necessary, signals do not have to be processed, which could introduce errors and requires supervision during the feature extraction phase.

One of the most remarkable results of this work is that the use of simulated ECGs for training not only predicts the OTVA SOO with good accuracy, but it even surpasses the performance of the databases trained with real data, especially when they are used with a different database. This is due to the higher variability of the simulated data, which also presents less bias towards acquisition instruments or protocol. Consequently, as it is shown in Scenario 3, the addition of simulated data to real databases can improve the prediction of the SOO in any dataset when compared with the results obtained by training with only real data (Scenario 2). These results support the use of simulated signals for improving the performance of ML classifiers, as it has been done previously for atrial fibrillation (Luongo et al., 2021) or cardiac resynchronization therapy response (Khamzin et al., 2021). On the other hand, the inclusion of augmented simulated data (DS-7488) did not had a significant impact in the classification results, probably due to the high correlation of the data. We also analyzed why the DS-31 dataset presents lower classification accuracies than DS–334. An analysis of the classification results (see Supplementary Material S1) showed that some of the LV SOO were wrongly classified as RV SOO. This was caused by some LV signals presenting variability that could not be reproduced by the simulated ECGs used for training. Furthermore, the reduced sample size of this dataset negatively affected the computed accuracies.

It is important to note that the designed ML-based pipeline does not require any further complex and time-consuming simulations, unless there is a need to update the model with additional data. That is one of the main limitations of physics-based approaches, in which the construction of the patient digital twin, and the computation of electrophysiology simulations is complex and requires hours to days to produce the results (Prakosa et al., 2018). This makes the ML-based approach suitable to be transferred to the clinical routine, since it can make instantaneous predictions with the only requirement of accessing the 12-lead ECG. This is a critical step towards the implantation of computational techniques for therapy planning of catheter-based ablation, since they can help to reduce procedure times, improve the risk evaluation or identify arrhythmias that cannot be treated (e.g. inaccessible SOOs (Yamada et al., 2010)) before the intervention. There have been previous works that made use of ML models to predict the SOO using only clinical data for training with good accuracy (0.97) at the cost of having a large feature vector of 1.6 million size (Zheng et al., 2021), which could show problems to generalize for other databases.

### 4.1 Limitations

Even though our method shows promising results when compared to existing solutions, it presents some limitations. First of all, to build the database, digital twin models must be faithful representations of patients, and the biophysical simulations have to be properly calibrated to produce realistic simulations that provide ECGs comparable to those recorded in clinical practice (Lopez-Perez et al., 2015). Otherwise, the training dataset could represent only a subset of the population and have problems generalizing to other datasets and patients. We are aware that having a single torso geometry, where all the personalized ventricular anatomies are registered could also be a limitation, since it has been reported that changes in the orientation of the heart or disposition of pericardial fat could have important effects in the ECG (Bradley et al., 2000; Gyawali et al., 2020). In our models, we have not considered the inclusion of a personalized Purkinje system, which could interact with the electrical sequence of activation (Sebastian et al., 2011; Cárdenes et al., 2015).

In addition, although we include variability in our simulations (different electrode location, SOO or digital twin anatomy), simulated data still is highly dependent on the initial conditions of the model. Increasing the number of the simulations, and varying additional parameters (new torso geometry, SOOs, different ectopic coupling interval, conduction velocity or heart rate) could reduce the bias that the simulated data may present. The use of more anatomies could help to cover a wider range of anatomical variability. In this study we used 16 patient-specific anatomies that presented considerable differences in shape and volume, but including a greater inter-subject variability could also improve the simulated ECG data. However, the computational time necessary to build or extend the simulated database can increase considerably.

Finally, we have not explored the classification of the SOO in the nine sublocations (e.g., LCC, RFW or AMC). The available datasets did not produced enough well-labeled data, and some of these sublocations were underrepresented (e.g., LCC-RCC commissure, AMC). This same limitation was present in other works that used similar datasets (Zheng et al., 2021). Richer clinical data for testing the ML models, together with more accessible OTVA datasets, will help in the prediction of the SOO with more accuracy.

## 5 Conclusion

We have shown a computational approach to predict the SOO of idiopathic ventricular tachycardia originated in the ventricular outflow tract. The method, that relies in biophysical simulation and machine learning techniques, is able to differentiate between LV or RV origin of the ectopic beat with an accuracy of 0.86 in a clinical database of 334 patients, and 0.84 in a second clinical database of 31 patients.

Since all the simulated training set was generated offline, the presented methodology could be transferred to a clinical environment, avoiding the need of time consuming tasks such as building computational models of the heart and performing electrophysiology simulations. Nevertheless, the simulated signals (DS-2496, DS-7488) achieved high performance in the test sets (DS-344 and DS-31), demonstrating the viability to produce good classification models for real data. Moreover, the methodology is not dependent on the expertise of the electrophysiologist, and it is consistent between cases, which could provide an additional tool to electrophysiologist to plan RFA interventions of this type of tachycardia. Future works will focus on improving the accurate determination of the exact SOO of the tachycardia within the ventricles, especially in the outflow tract sublocations.

## Data Availability

The raw data supporting the conclusion of this article will be made available by the authors, without undue reservation.
