# Supplementary material for: Training machine learning models with synthetic data improves the prediction of ventricular origin in outflow tract ventricular arrhythmias
Source: Front Physiol. 2022 Aug 12;13:909372. doi: 10.3389/fphys.2022.909372 (PMC9412034; doi:10.3389/fphys.2022.909372)
Supplement: Supplementary file 1 [file DataSheet1.PDF]

Supplementary Material

1 Classification Reports

Confusion matrix and classification reports for the main classification Scenarios:

1.1 Scenario 1 (Raw Data)

<u>Test: DS-334</u>

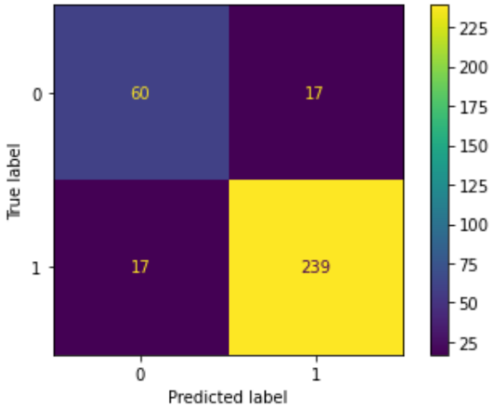

	precision	recall	f1-score	support
LV	0.78	0.78	0.78	77
RV	0.93	0.93	0.93	256
accuracy			0.90	333
macro avg	0.86	0.86	0.86	333
weighted avg	0.90	0.90	0.90	333

<u>Test: DS-31</u>

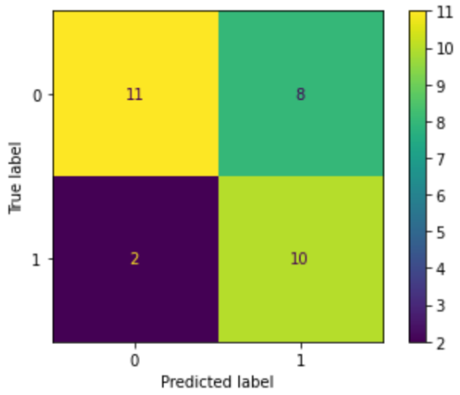

	precision	recall	f1-score	support
LV	0.85	0.58	0.69	19
RV	0.56	0.83	0.67	12
accuracy			0.68	31
macro avg	0.70	0.71	0.68	31
weighted avg	0.73	0.68	0.68	31

1.2 Scenario 1 (Featured Data)

<u>Test: DS-334</u>

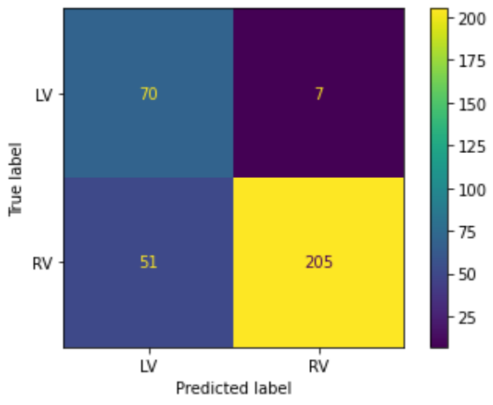

	precision	recall	f1-score	support
LV	0.58	0.91	0.71	77
RV	0.97	0.80	0.88	256
accuracy			0.83	333
macro avg	0.77	0.85	0.79	333
weighted avg	0.88	0.83	0.84	333

<u>Test: DS-31</u>

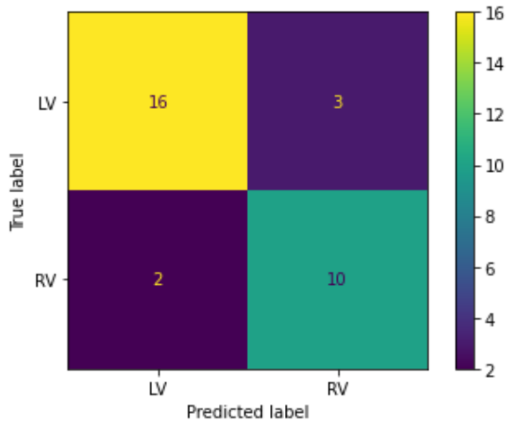

	precision	recall	f1-score	support
LV	0.89	0.84	0.86	19
RV	0.77	0.83	0.80	12
accuracy			0.84	31
macro avg	0.83	0.84	0.83	31
weighted avg	0.84	0.84	0.84	31

1.3 Scenario 2 (Raw Data)

Training: DS-334 Test: DS-31

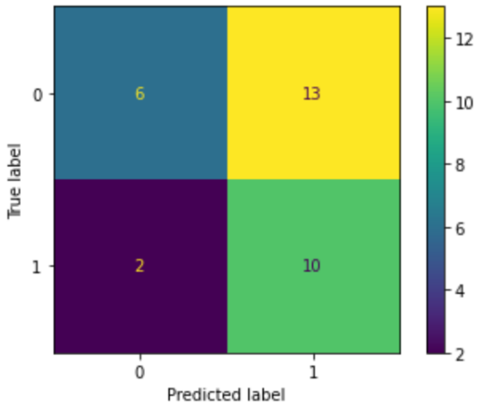

	precision	recall	f1-score	support
LV	0.75	0.32	0.44	19
RV	0.43	0.83	0.57	12
accuracy			0.52	31
macro avg	0.59	0.57	0.51	31
weighted avg	0.63	0.52	0.49	31

Training: DS-31 Test: DS-334

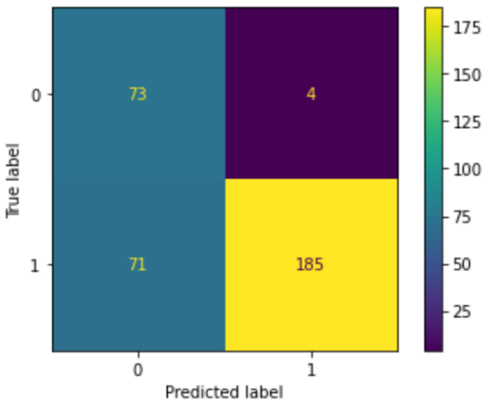

	precision	recall	f1-score	support
LV	0.51	0.95	0.66	77
RV	0.98	0.72	0.83	256
accuracy			0.77	333
macro avg	0.74	0.84	0.75	333
weighted avg	0.87	0.77	0.79	333

1.4 Scenario 2 (Featured Data)

Training: DS-334 Test: DS-31

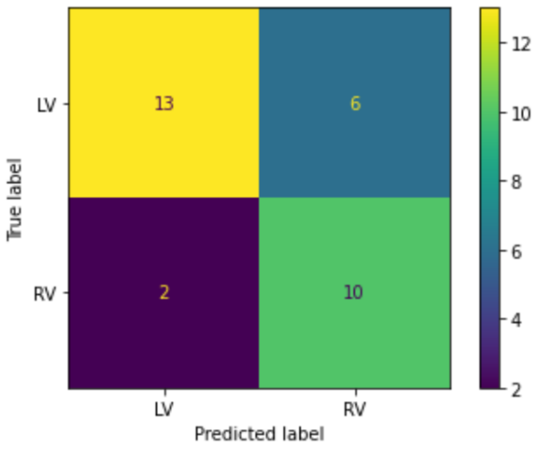

	precision	recall	f1-score	support
LV	0.87	0.68	0.76	19
RV	0.62	0.83	0.71	12
accuracy			0.74	31
macro avg	0.75	0.76	0.74	31
weighted avg	0.77	0.74	0.75	31

Training: DS-31 Test: DS-334

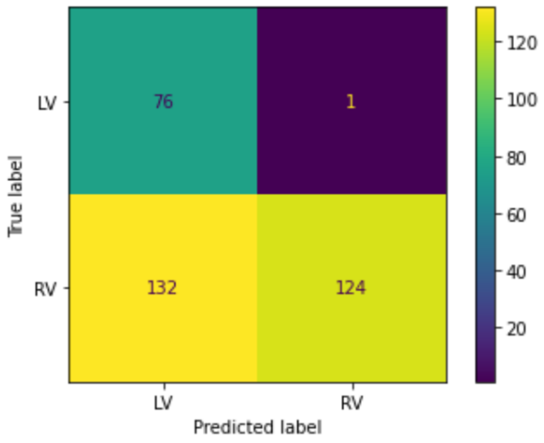

	precision	recall	f1-score	support
LV	0.37	0.99	0.53	77
RV	0.99	0.48	0.65	256
accuracy			0.60	333
macro avg	0.68	0.74	0.59	333
weighted avg	0.85	0.60	0.62	333

2 Signal Comparison

The Leads of the different signals from the datasets were compared using the maximum mean discrepancy (MMD), as proposed in previous works [1]. Two different comparisons were carried out, with (top row) and without (bottom row) normalizing the data.

Lead	MMD (DS-31 vs. DS-334)	MMD (DS-31 vs. DS-2496)	MMD (DS-334 vs. DS-2496)	MMD (DS-31 vs. DS-7488)	MMD (DS-334 vs. DS-7488)	MMD (DS-2496 vs. DS-7488)
I	0.029244	0.031940	0.031480	0.016649	0.038365	0.022549
II	0.000447	0.001795	0.002340	0.000411	0.000418	0.001871
III	0.000352	0.001898	0.002716	0.000478	0.000538	0.002031
AVR	0.012506	0.014931	0.016808	0.003067	0.004095	0.006518
AVL	0.004467	0.012996	0.023712	0.003596	0.005839	0.010333
AVF	0.000993	0.002010	0.003198	0.000498	0.000680	0.002008
V1	0.000217	0.000402	0.000811	0.000325	0.000511	0.000548
V2	0.000000	0.000023	0.000024	0.000080	0.000079	0.000101
V3	0.000003	0.000061	0.000081	0.000182	0.000189	0.000292
V4	0.000033	0.000467	0.000670	0.000345	0.000379	0.001153
V5	0.000110	0.000937	0.001820	0.000658	0.000882	0.001982
V6	0.000404	0.004814	0.009242	0.001784	0.002965	0.008091
Mean	0.004065	0.006023	0.007742	0.002339	0.004578	0.00479

Lead	Normalized MMD (DS-31 vs. DS-334)	Normalized MMD (DS-31 vs. DS-2496)	Normalized MMD (DS-334 vs. DS-2496)	Normalized MMD (DS-31 vs. DS-7488)	Normalized MMD (DS-334 vs. DS-7488)	Normalized MMD (DS-2496 vs. DS-7488)
I	0.014109	0.036114	0.027302	0.045085	0.035794	0.005558
II	0.000449	0.001977	0.002107	0.001968	0.001837	0.001719
III	0.000266	0.002096	0.002370	0.002437	0.002375	0.001904
AVR	0.013514	0.025416	0.013497	0.032498	0.018539	0.005101
AVL	0.002792	0.017036	0.019884	0.028760	0.030151	0.006075
AVF	0.000530	0.002161	0.002684	0.002462	0.002561	0.001921
V1	0.000347	0.000899	0.000607	0.001656	0.001329	0.000742
V2	0.000003	0.000028	0.000024	0.000504	0.000468	0.000351
V3	0.000002	0.000083	0.000080	0.000791	0.000783	0.000657
V4	0.000002	0.000620	0.000661	0.001526	0.001606	0.001350
V5	0.000039	0.001288	0.001442	0.004036	0.004486	0.003029
V6	0.000080	0.007866	0.006394	0.015226	0.013317	0.006987
Mean	0.002678	0.007965	0.006421	0.011412	0.009437	0.002949

Table S1: Signal comparison between the different datasets used in the classification. MMD: maximum mean discrepancy.

[1] Delaney, A. M., E. Brophy, and T. E. Ward. "Synthesis of Realistic ECG using Generative Adversarial Networks. arXiv 2019." arXiv preprint arXiv:1909.09150.
